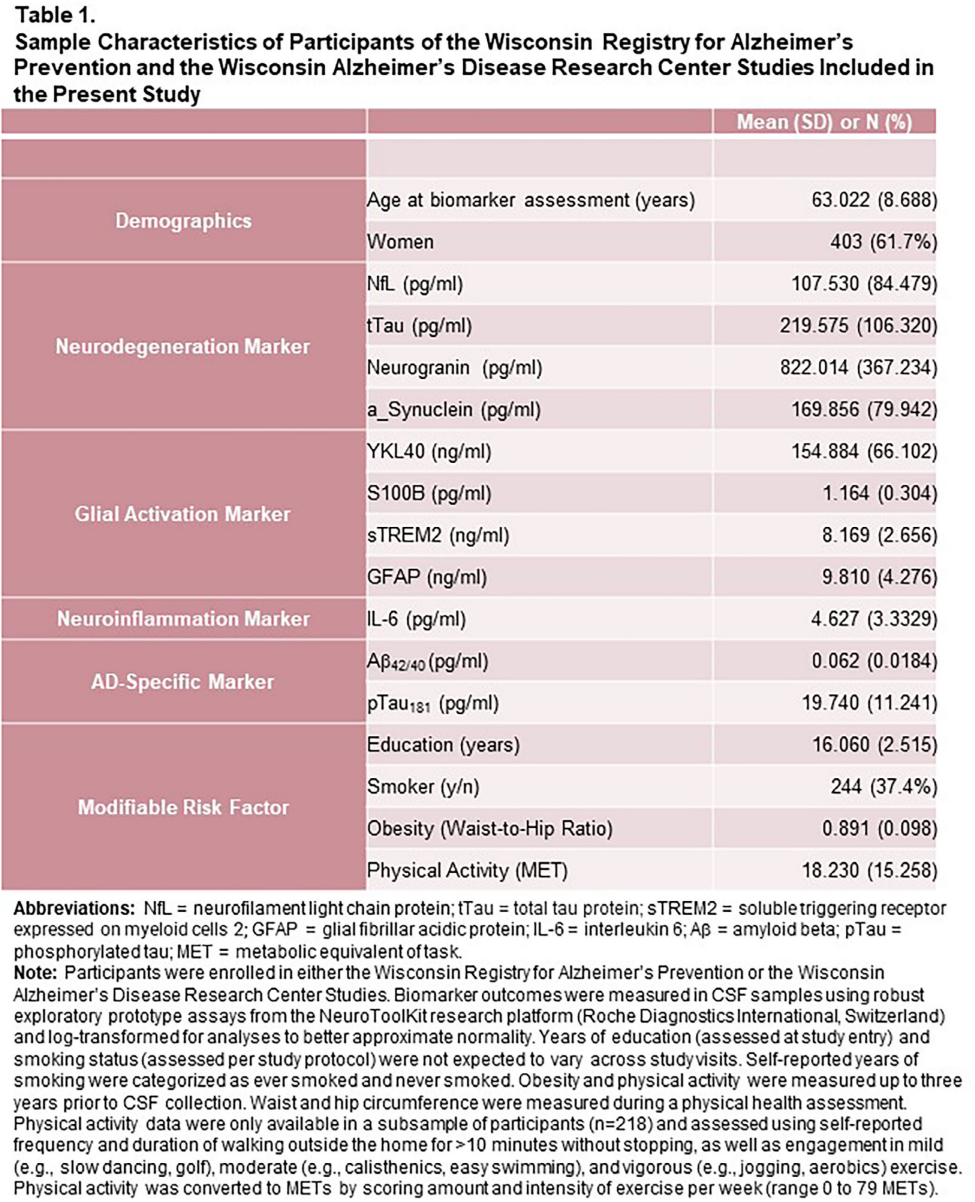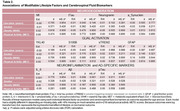# Associations of education and modifiable lifestyle factors with CSF biomarkers of neurodegeneration, neuroinflammation, and Alzheimer’s disease

**DOI:** 10.1002/alz.091653

**Published:** 2025-01-09

**Authors:** Beth M. Planalp, Carol A. Van Hulle, Barbara B. Bendlin, Kaj Blennow, Clara Quijano‐Rubio, Corinne D. Engelman, Gwendlyn Kollmorgen, Yue Ma, Ozioma C Okonkwo, Cynthia M. Carlsson, Sterling C. Johnson, Henrik Zetterberg, Natascha Merten

**Affiliations:** ^1^ University of Wisconsin‐Madison, Madison, WI USA; ^2^ Wisconsin Institute for Alzheimer’s, School of Medicine and Public Health, University of Wisconsin—Madison, Madison, WI USA; ^3^ Wisconsin Alzheimer’s Institute, University of Wisconsin‐Madison, School of Medicine and Public Health, Madison, WI USA; ^4^ Wisconsin Alzheimer's Disease Research Center, University of Wisconsin School of Medicine and Public Health, Madison, WI USA; ^5^ Geriatric Research Education and Clinical Center, William S. Middleton Memorial Veterans Hospital, Madison, WI USA; ^6^ Wisconsin Alzheimer's Institute, University of Wisconsin School of Medicine and Public Health, Madison, WI USA; ^7^ Clinical Neurochemistry Laboratory, Sahlgrenska University Hospital, Mölndal Sweden; ^8^ Department of Psychiatry and Neurochemistry, Institute of Neuroscience and Physiology, the Sahlgrenska Academy at the University of Gothenburg, Mölndal Sweden; ^9^ Roche Diagnostics International Ltd., Rotkreuz Switzerland; ^10^ University of Wisconsin‐Madison, School of Medicine and Public Health, Madison, WI USA; ^11^ Roche Diagnostics GmbH, Penzberg Germany; ^12^ University of Wisconsin, Madison, WI USA; ^13^ Division of Geriatrics, Department of Medicine, University of Wisconsin School of Medicine and Public Health, Madison, WI USA; ^14^ Geriatric Research, Education and Clinical Center (GRECC), William S. Middleton Memorial Veterans Hospital, Madison, WI USA; ^15^ Division of Geriatrics and Gerontology, Department of Medicine, University of Wisconsin School of Medicine and Public Health, Madison, WI USA; ^16^ UK Dementia Research Institute, University College London, London UK; ^17^ Department of Population Health Sciences, School of Medicine and Public Health, University of Wisconsin‐Madison, Madison, WI USA; ^18^ Wisconsin Alzheimer’s Disease Research Center, School of Medicine and Public Health, University of Wisconsin‐Madison, Madison, WI USA

## Abstract

**Background:**

Alzheimer’s disease (AD) and related dementias can have long preclinical phases; thus, midlife intervention and prevention methods could prove efficacious. Multiple health‐related lifestyle factors have been associated with risk for AD. However, research on lifestyle factors has focused on clinical outcomes such as cognitive decline, mild cognitive impairment and/or AD dementia; their associations with potential early changes in cerebrospinal fluid (CSF) biomarkers are less understood. Our aim was to determine whether key lifestyle factors are associated with CSF biomarkers for neurodegeneration, glial activation, and neuroinflammation.

**Method:**

Participants were N=653 adults (aged 43—93 years) from the Wisconsin Registry for Alzheimer’s Prevention and the Wisconsin Alzheimer’s Disease Research Center, two on‐going, longitudinal studies of preclinical AD. We assessed CSF biomarkers using the NeuroToolKit (NTK), a panel of robust prototype biomarker assays (Roche Diagnostics International, Switzerland). Participants completed demographic and health questionnaires and a physical assessment (Table 1). Linear regression models assessed standardized effects of associations between lifestyle factors (education, smoking, obesity, and physical activity) and CSF biomarkers, adjusting for age and sex.

**Result:**

After correcting for false discovery rate, preliminary results (Table 2) showed that participants with more years of education had higher CSF Aβ_42/40_ and lower pTau_181_ concentrations (Est. = 0.006, or 1 unit increase in Aβ_42/40_ per year increase in education, 95% confidence interval: 0.001–0.011; p =.008, adjusted p = .061; Est.= ‐0.007, or 1 unit decrease in pTau_181_ per year increase in education, 95% confidence interval: ‐0.013–‐0.002; p=.026, adjusted p=.061). No other biomarker associations withstood correction for multiple comparisons.

**Conclusion:**

Education has been discussed as an early modifiable risk factor for AD, likely through a mechanism of cognitive resilience. We found that higher education was associated with less AD proteinopathy (e.g. better Ab_42/40_ and pTau_181_ profiles). Future studies should explore the potential underlying mechanisms for this association. Longer follow‐up will help to understand how these findings relate to biomarker levels and cognitive changes at a later stage of disease progression.